# Human Somatostatin SST_4_ Receptor Transgenic Mice: Construction and Brain Expression Pattern Characterization

**DOI:** 10.3390/ijms22073758

**Published:** 2021-04-04

**Authors:** Balázs Nemes, Kata Bölcskei, Angéla Kecskés, Viktória Kormos, Balázs Gaszner, Timea Aczél, Dániel Hegedüs, Erika Pintér, Zsuzsanna Helyes, Zoltán Sándor

**Affiliations:** 1 Department of Pharmacology and Pharmacotherapy, University of Pécs Medical School & Centre for Neuroscience, H-7624 Pécs, Hungary; balazs.nemes@aok.pte.hu (B.N.); kata.bolcskei@aok.pte.hu (K.B.); angela.kecskes@aok.pte.hu (A.K.); viktoria.kormos@aok.pte.hu (V.K.); aczel.timea@pte.hu (T.A.); erika.pinter@aok.pte.hu (E.P.); zoltan.sandor@aok.pte.hu (Z.S.); 2 János Szentágothai Research Centre, University of Pécs, H-7624 Pécs, Hungary; 3 Research Group for Mood Disorders, Department of Anatomy, University of Pécs Medical School & Centre for Neuroscience, H-7624 Pécs, Hungary; balazs.b.gaszner@aok.pte.hu (B.G.); hedani93@gmail.com (D.H.); 4 PharmInVivo Ltd., H-7629 Pécs, Hungary; 5 Algonist Biotechnologies Gmbh, 1030 Wien, Austria

**Keywords:** somatostatin, *Sstr4* KO mice, *SSTR4* humanized mice, PiggyBac (PB) transposon vector, ligation-mediated PCR, RNAscope in situ hybridization

## Abstract

Somatostatin receptor subtype 4 (SST_4_) has been shown to mediate analgesic, antidepressant and anti-inflammatory functions without endocrine actions; therefore, it is proposed to be a novel target for drug development. To overcome the species differences of SST_4_ receptor expression and function between humans and mice, we generated an SST_4_ humanized mouse line to serve as a translational animal model for preclinical research. A transposon vector containing the *hSSTR4* and reporter gene construct driven by the *hSSTR4* regulatory elements were created. The vector was randomly inserted in *Sstr4*-deficient mice. *hSSTR4* expression was detected by bioluminescent in vivo imaging of the luciferase reporter predominantly in the brain. RT-qPCR confirmed the expression of the human gene in the brain and various peripheral tissues consistent with the in vivo imaging. RNAscope in situ hybridization revealed the presence of *hSSTR4* transcripts in glutamatergic excitatory neurons in the CA1 and CA2 regions of the hippocampus; in the GABAergic interneurons in the granular layer of the olfactory bulb and in both types of neurons in the primary somatosensory cortex, piriform cortex, prelimbic cortex and amygdala. This novel SST_4_ humanized mouse line might enable us to investigate the differences of human and mouse SST_4_ receptor expression and function and assess the effects of SST_4_ receptor agonist drug candidates.

## 1. Introduction

Somatostatin is a cyclic neuropeptide, which inhibits the secretion of several excitatory and inhibitory mediators, such as somatotropin, glucagon, insulin, acetylcholine, glutamate and gamma-aminobutyric acid (GABA) [1]. It regulates a range of physiological functions like sleep, motor activity, sensory functions, emotions, learning and memory, as well as different pathological conditions like pain, inflammation [2,3,4,5], neurodegeneration [6,7,8,9], anxiety and depression [10,11,12,13]. In the central nervous system, there are long protruding and short proximal somatostatin-containing GABAergic interneurons [14,15,16]. In the periphery, activated capsaicin-sensitive peptidergic sensory nerves were observed to release somatostatin and induce systemic anti-inflammatory and analgesic effects, which was termed “sensocrine” action [17,18]. Five G_i_-protein-coupled receptors, designated SST_1_-SST_5_, mediate the actions of somatostatin. Previous work provided evidence that the SST_4_ receptor activation produces analgesic, anti-inflammatory, anti-amyloid, anti-anxiety and antidepressant effects without influencing hormone secretion [2,19,20,21,22,23].

Previous studies used *Sstr4* knockout (KO) and wild-type (WT) mice, since no SST4 receptor specific antagonist is commercially available. Investigating *Sstr4* KO mouse models and the synthetic SST_4_ receptor agonist J-2156, we and other workgroups provided evidence that the SST_4_ receptor is a unique novel drug target for the treatment of chronic pain and depression [2,4,20,24,25,26,27].

The presently available drugs for these conditions are often not efficacious enough and cause serious side effects upon long-term use [28]. Thus, the SST_4_ receptor has recently come into the focus of interest of drug development, and pharmaceutical companies have started to develop non-peptide SST_4_ agonists [29]. The agonist design has been greatly enhanced by the in silico 3D modeling of the human receptor structure [30,31].

Humanized mice are predominantly generated by engrafting human cells, tissues and tumors and used extensively for immunological and oncological research [32,33,34,35,36,37], but genetic modification is also an increasingly applied method [38,39,40,41]. Human receptor expressing mouse models are useful in translational drug research, providing more predictive results relevant for the human diseases and more relevant platforms for testing drug candidates [42,43]. For example, Fox et al. replaced the native bradykinin B_1_ receptor gene in mouse with its human B_1_ receptor gene to investigate the human B_1_ receptor-specific antagonist NVP-SAA164 in vivo after the successful in vitro results. NVP-SAA164 showed an anti-hyperalgesic function in these humanized mice, but not in WT nor in KO mice [44]. Jackson et al. found functional differences between mouse and human melanocortin receptor using MC1R humanized mice, such as the high ligand dependent eumelanogenesis in humanized mice. Mouse Mc1r receptor in WT mice in vivo, and both mouse and human melanocortin receptors in transfected cell lines in vitro showed ligand independent signaling [45].

The mouse SST_4_ expression and function is relatively well-characterized in the brain [46], but little is known about the human receptor.

Therefore, our aims were to produce humanized mice by using a transposon vector containing the *hSSTR4* gene with its regulatory elements, locate the random insertion sites and characterize the distribution pattern and the neuron populations expressing the transgene in the brain, as well as select the most useful mouse line for further functional research.

## 2. Results

### 2.1. Vector Construction and Transgenesis

**Figure 1:**
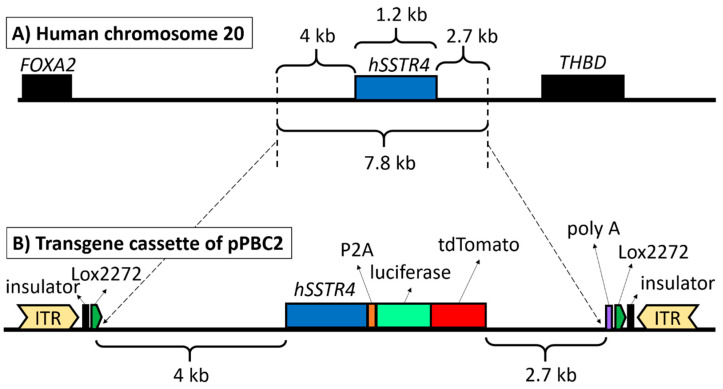
The structure of the PiggyBac (PB) transposon. Human chromosome 20 showing the full-length *hSSTR4* coding sequence with up- and downstream regulatory elements (7.8 kb) as copied fragments and the neighboring genes (*FOXA2* and *THBD*) (**A**). PB transposon vector carrying the upstream regulatory elements (4 kb) and human *SSTR4* coding sequence followed by the P2A self-cleaving peptide sequence, luciferase and tdTomato coding sequences downstream *SSTR4* regulatory elements (2.7 kb) and polyadenylation signal sequence (poly A). The entire transgenic cassette (12.7 kb) was flanked by the PB transposon inverted terminal repeats (ITR), insulators and Lox2272 Cre recombinase recognition sites (**B**).

The final construct pPBC2 plasmid contained the full-length *hSSTR4* gene with a 4-kb upstream and 2.7-kb downstream region of the human chromosome 20 to include every known regulatory element (Figure 1A). At the end of the *hSSTR4* coding sequence, luciferase and the tandem dimer Tomato (tdTomato) coding sequence were inserted, separated from the receptor by the P2A self-cleaving site, but the reporters were expected to be translated as a fusion protein. At the end of the downstream chromosomal region, a polyadenylation signal (polyA) sequence was added for transcription termination. The entire transgene cassette was flanked at both ends by Lox2272 Cre recombinase recognition sites as an option for conditional KO by insulators to inhibit position effects and by PiggyBac (PB) transposon inverted terminal repeats (ITR) as specific recognition sites for the PB transposase enzyme (Figure 1B).

**Figure 2:**
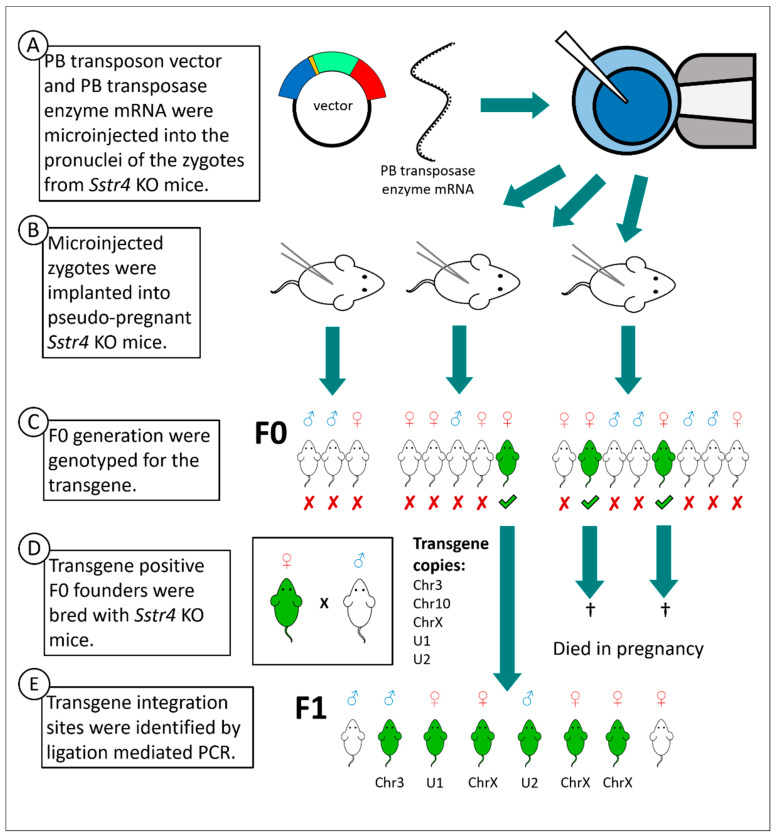
Steps of transgenesis by PB transposon vector. Schematic generation of *hSSTR4* transgenic mice (**A**–**E**). White mice symbolize *Sstr4* knockout (KO) mice lacking *hSSTR4* transgene, green mice symbolize the correctly inserted *hSSTR4* transgene. The transgene copies are named Chr3, Chr10 and ChrX (located in chromosomes 3, 10 and X, respectively) and U1 and U2 (the two copies in an unknown location).

The vector was verified by sequencing as successfully constructed before it was injected into 56 *Sstr4* KO zygotes, along with the PB transposase mRNA (Figure 2A). After injection, the intact zygotes were implanted into three pseudo-pregnant *Sstr4* KO mice (Figure 2B). Sixteen mice were born in the F0 generation, out of which three female mice were verified by PCR carrying the PB transposon without the plasmid backbone (Figure 2C). These transgene positive mice were crossbred with *Sstr4* KO mice. Two females died in late pregnancy. The third female produced 8 offspring (F1 generation), but it also died during its second pregnancy (Figure 2D). In this founder mouse, the PB transposon integration sites were located by ligation-mediated polymerase chain reaction (LM-PCR) (Figure 2E). From the F1 generation onwards, all mouse lines carried a single copy of the transgene, and none of those lines manifest a harmful phenotype based on viability, fertility and behavior. There were no differences between the litter sizes of the mouse lines (WT: 6.2 ± 0.4, KO: 5.8 ± 0.3, Chr3: 6.5 ± 0.4, U1: 6.0 ± 0.4, and U2: 5.9 ± 0.4).

### 2.2. Integration Sites of the hSSTR4 Transgene

**Figure 3:**
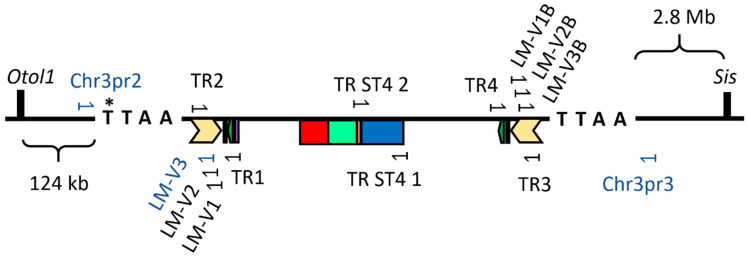
The insertion site of the PB transposon in chromosome 3 of the *Sstr4* KO mouse genome, along with the primers used for genotyping. Mouse chromosome 3 showing the location of the transgene insertion in the PB transposase recognition site (TTAA) at the original position of 70,039,120th base pair (*), which was located between the neighboring genes (*Otol1* and *Sis*). Primer sites are shown in the transgene, which integrated in the chromosome 3 used for the first genotyping test of the F0 generation (TR1-4), for verifying the sequencing of the *hSSTR4* coding sequence (TR ST4 1–2), for ligation-mediated PCR (LM-V primers) and for present routine genotyping PCR (LM-V3 and Chr3pr2-3, marked with blue).

The LM-PCR detected multiple insertion loci of the transgene in the F0 founder female. By sequencing of the LM-PCR products, three distinct integration sites were identified in chromosomes 3, 10 and X (copies were named Chr3, Chr10 and ChrX, respectively). The transgene inserted in the chromosome 10 did not pass onto the F1 generation (Figure 2). The transgene in the chromosome 3 was inserted between the *Sis* and *Otol1* genes, coding on the negative strand of the chromosome (Figure 3). The PB transposase inserted the transposon while duplicating the TTAA recognition site, which started in the original position of 70,039,120 [47] (Figure 3). The transgene inserted in the chromosome 10 in the position of 67,335,940 [48], coding on the positive strand. The transgene inserted in the chromosome X at the position of 32,102,395 [49], coding on the positive strand. There were two mouse lines in which we were unable to locate the insertion site of the transgene (named U1 and U2) (Figure 2).

To keep track of the transgene in the mouse lines, we designed site-specific genotyping assays for each copy in a known location, and we could differentiate hetero- and homozygotes. In the case of U1 and U2, we could only detect the presence of the transgene, but we could not distinguish the hetero- and homozygotes; therefore, we bred them strictly with *Sstr4* KO mice to obtain only heterozygous mice for testing.

### 2.3. Distinct Expression Pattern of the hSSTR4-Related Luciferase by Luminescent In Vivo Optical Imaging in Various Mouse Lines

**Figure 4:**
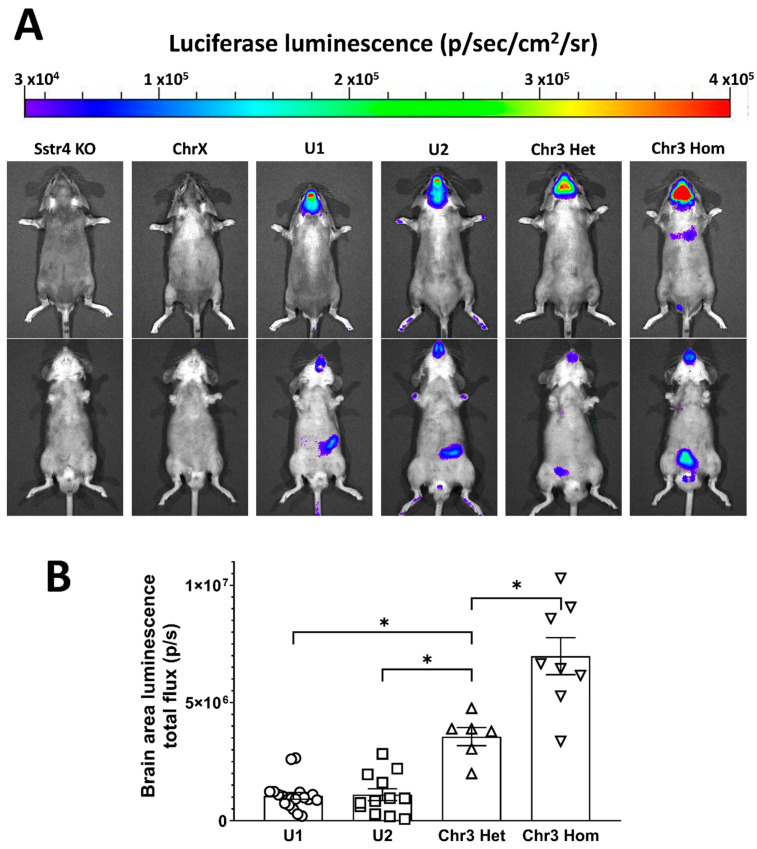
In vivo bioluminescent imaging of the luciferase reporter protein co-expressed with the *hSSTR4* gene. Representative images show the differences of expression pattern and luminescence intensity of luciferase in the different mouse lines (**A**). Scatter plot with bars show the means ± SEM with the individual data points of the luminescence intensity in equal size areas of the head corresponding to the brain (**B**). One-way ANOVA, * *p* < 0.0001, *N* = 6–19/genotype.

First, we characterized the expression of the transgene by measuring the signals of the co-expressed reporter fusion protein: the luminescence of the luciferase and the fluorescence of the tdTomato. Luciferase activity was visualized in the whole body (Figure 4A) and quantified in regions of interest (ROI) in the area of the brain (Figure 4B) by IVIS Lumina III. No luciferase activity was detected either in *Sstr4* KO mice or in hemizygote male or homozygote female ChrX *hSSTR4*-positive mice. Therefore, we excluded the ChrX line from later research, since the transgene was not expressed. Copies in unknown loci (U1 and U2) showed similar expression patterns: weak signal in the extremities and tail, medium signal strength in the area of cerebrum and the caudal part of the brain and strongest signal in the area of olfactory bulb (OB). Chr3 mice showed the strongest signal in the area corresponding to the cerebral hemispheres. Chr3 homozygous mice had nearly exactly double the signal intensity compared to Chr3 heterozygous mice. Chr3 Het mice had significantly stronger signal than U1 and U2 (also heterozygous) mice (Figure 4B). In the periphery, the luminescent signal was weaker and variable, with the highest levels in the lower abdomen and pelvis, regardless of the chromosomal location of the copy of the transgene. Fluorescence of tdTomato linked to *hSSTR4* was not detectable in vivo by optical imaging (image is not shown).

### 2.4. hSSTR4 Expression Level Assessed by RT-qPCR

**Figure 5:**
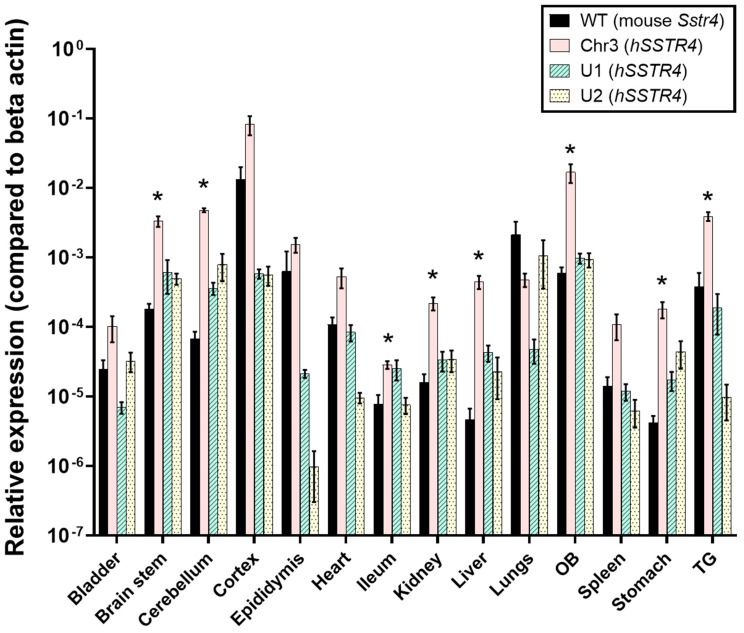
Relative mouse *Sstr4* and human *SSTR4* mRNA expression levels in wild-type (WT) and humanized (Chr3, U1 and U2) mice, respectively. The diagram shows RT-qPCR results (2^−ΔCt^) relative to the beta actin (*Actb*) mRNA reference gene in the tested organs. Cortex, OB and TG stand for cerebral cortex, olfactory bulb and trigeminal ganglion, respectively. Each column shows the mean ± SEM. The significant differences between the WT and Chr3 mice are indicated with an asterisk above the Chr3 column. Kruskal-Wallis test with Dunn’s post-test; * *p* < 0.01; *N* = 3–7/genotype.

In every inspected organ, with the exception of the lungs, Chr3 showed a higher expression of *hSSTR4* than the mouse *Sstr4* expression in the WT mice. Chr3 showed the highest *hSSTR4* expression in the nervous system: cerebral cortex, olfactory bulb (OB), trigeminal ganglia (TG), cerebellum and brain stem, and also a relatively high expression in the epididymis. In comparison, the mouse *Sstr4* expression in the WT mice was the highest in the cerebral cortex, lungs, OB, epididymis and TG but much lower in the brain stem and cerebellum than the humanized mice. Generally, U1 and U2 showed lower expression levels of *hSSTR4* in every organ, with the exception of the lungs, compared to Chr3 mice. (Figure 5).

### 2.5. Expression of hSSTR4 mRNA in Different Types of Neurons in the Brain

**Figure 6:**
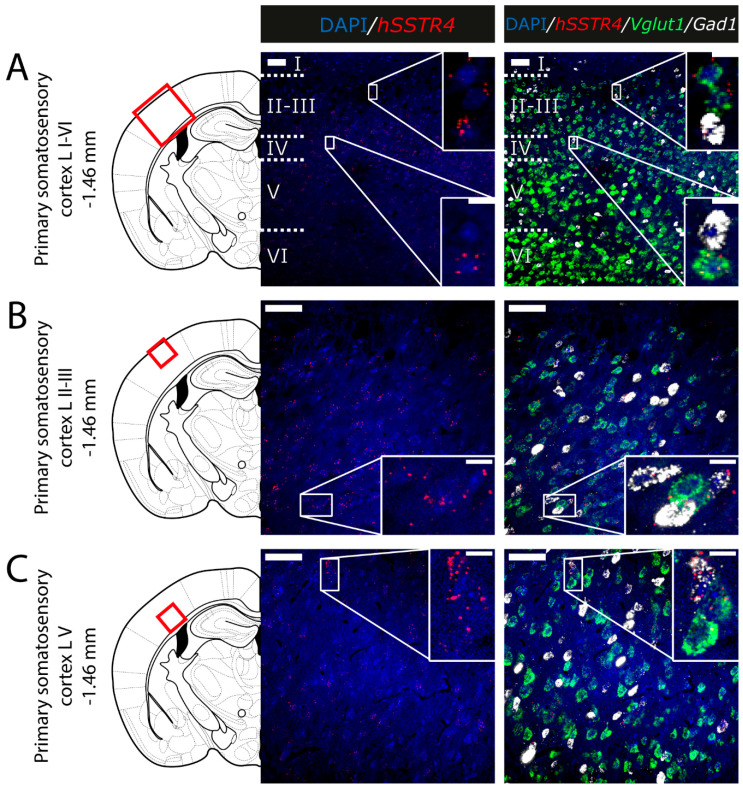
Expression of the *hSSTR4* mRNA in the mouse primary somatosensory cortex. Representative confocal images from Chr3 homozygote mice. Human *SSTR4* (red), mouse *Vglut1* (green) and mouse *Gad1* (white) mRNA expressions counterstained with 4′,6-diamidino-2-phenylindole (DAPI) (blue) were shown in the layer I-VI (**A**), the layer II-III (**B**) and the layer V (**C**) of the primary somatosensory cortex (S1, Bregma −1.46 mm). Scale bar: 50 µm, and inset scale bar: 10 µm.

**Figure 7:**
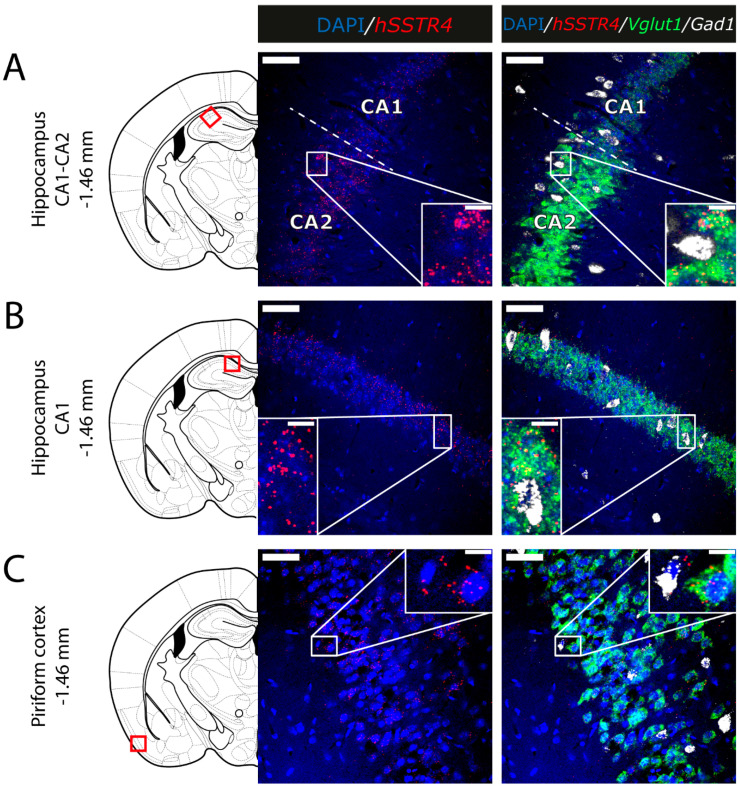
Expression of the *hSSTR4* mRNA in the mouse hippocampus and piriform cortex. Representative confocal images from Chr3 homozygote mice. Human *SSTR4* (red), mouse *Vglut1* (green) and mouse *Gad1* (white) mRNA expressions counterstained with DAPI (blue) were shown in the CA1-CA2 region (Bregma −1.46 mm, (**A**)), CA1 region of the hippocampus (Bregma −1.46 mm, (**B**)) and the piriform cortex (Pir, Bregma −1.46 mm, (**C**)). Scale bar: 50 µm, and inset scale bar: 10 µm.

**Figure 8:**
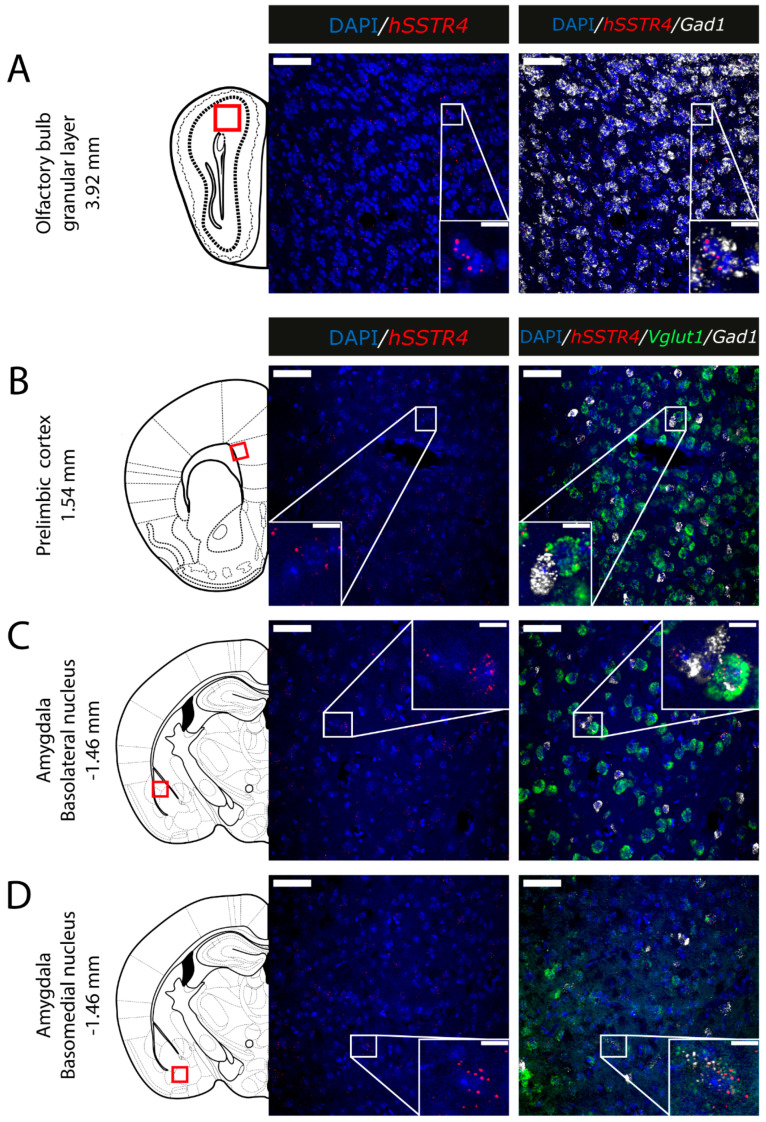
Expression of the *hSSTR4* mRNA in the mouse olfactory bulb, prelimbic cortex and amygdala. Representative confocal images from Chr3 homozygote mice. Human *SSTR4* (red), mouse *Vglut1* (green) and mouse *Gad1* (white) mRNA expressions counterstained with DAPI (blue) were shown in the granular layer of the olfactory bulb (OB, Bregma +3.92 mm, (**A**)), prelimbic cortex (PrL, Bregma +1.54 mm, (**B**)), basolateral (BLA, (**C**)) and basomedial (BMA, (**D**)) nucleus of the amygdala (Bregma −1.46 mm). Scale bar: 50 µm, and inset scale bar: 10 µm.

**Figure A1:**
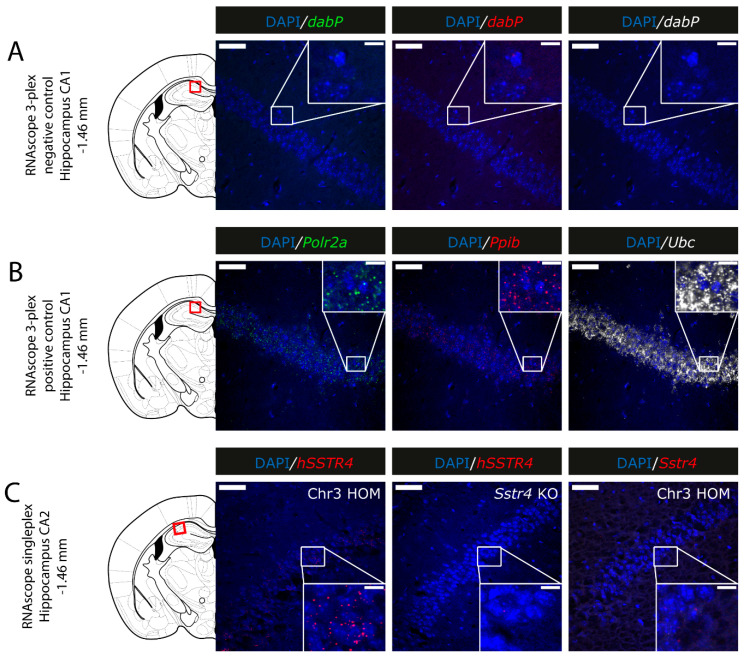
Representative control conditions for the RNAscope (counterstained with DAPI). RNAscope 3-plex-negative control specific to the bacterial *dabP* gene shown in the CA1 of *hSSTR4* homozygote mice (**A**). RNAscope 3-plex-positive control probes specific to mouse *Polr2a*, *Ppib* and *Ubc* mRNA targets shown in green, red and white, respectively, in the CA1 (Bregma −1.46 mm) of *hSSTR4* Chr3 homozygous animals (**B**). Human *SSTR4* singleplex control shown in the CA2 (Bregma −1.46 mm) of *hSSTR4* homozygous mice ((**C**), left panel). As negative tissue controls (no human *SSTR4* transcript was detected in *Sstr4* KO mice ((**C**), middle panel). Additionally, no mouse *Sstr4* signal was shown in *hSSTR4* homozygote mice ((**C**), right panel). Scale bar: 50 µm, and inset scale: 10 µm.

Since the *hSSTR4*-related tdTomato fluorescence was not detectable in native brain sections either (data not shown), and there is no reliably specific anti-SST_4_ antibody on the market, in situ hybridization of the *hSSTR4* mRNA was performed to explore its expression pattern. In brain samples of the *hSSTR4* Chr3 homozygous mice, the *hSSTR4* transcript showed a moderate expression level. Within the primary somatosensory cortex (S1), the highest level of expression was found in layers II and III (Figure 6A,B). The highest *hSSTR4* expression was detected in the CA2 field of the hippocampus and in the piriform cortex (Figure 7). Other areas of the brain, such as the granular layer of the OB, the prelimbic cortex, the basolateral (BLA) and the basomedial (BMA) nucleus of the amygdala, showed low, but still considerable *hSSTR4* expression levels (Figure 8). *hSSTR4* was predominantly localized on glutamatergic excitatory neurons (*Vglut1*) with inconsistent cases in the GABAergic interneurons (*Gad1*) in the primary somatosensory cortex (Figure 6), piriform cortex (Figure 7C), prelimbic cortex (Figure 8B), BLA (Figure 8C) and BMA (Figure 8D). In the hippocampus, CA1 and CA2 *hSSTR4* were localized in the glutamatergic excitatory neurons (Figure 7A,B). In the granular layer of the OB, *hSSTR4* transcripts were detected in the GABAergic interneurons (Figure 8A). See the control samples in Appendix A (Figure A1).

## 3. Discussion

The main impacts of the present work are that we successfully generated the first *hSSTR4* transgenic mouse line via random insertion of a PB transposon vector and characterized the receptor-expressing neurons in pain and mood regulation-related brain regions. These mice can be useful tools for preclinical research of the SST_4_ receptor, which is a promising novel target for analgesic and antidepressant drug development [2,22,23].

We chose random insertion instead of the popular knock-in approach, because we aimed to avoid the influence of mouse regulatory elements of the *Sstr4* gene on the transgene. We used a transposon vector with the intact human regulatory elements flanked by insulator regions to resemble the human receptor expression pattern. However, the disadvantage of this technique is that the mapping of the integration sites can be problematic, and the insert may disrupt mouse genes [50,51].

The random insertion of the PB transposon resulted in multiple integration sites in the F0 mouse generation. We successfully located three copies of the transgene by LM-PCR [52]: Chr3, Chr10 and ChrX, but the insertion sites of two copies (U1 and U2) are still unknown. The unsuccessful attempts to locate the integration sites of U1 and U2 suggest that they were inserted in repeat regions of the genome that makes mapping of the transgene difficult. Due to the known location of the transgene in the Chr3 mouse line, we could design site-specific genotyping assay to distinguish hetero- and homozygous mice.

All three transgenic mice in the F0 generation experienced complications during pregnancy and delivery and eventually died. This might have been due to the multicopy insertion of the transgene and the consequent overexpression of SST_4_, because this problem never occurred again in the offspring carrying a single copy. This observation also supports a role of SST_4_ in embryonal growth regulation, since it was demonstrated to be the predominant somatostatin receptor in the human placenta [53,54].

Bioluminescent in vivo optical imaging showed *hSSTR4-*linked luciferase expression pattern in different organs, and quantification showed that the luminescent signal was the strongest in the area corresponding to the brain. Chr3 mice showed a high expression in the area of the cerebrum, whereas U1 and U2 showed a lower expression here but a higher expression in the area of the OB and the caudal part of the brain. RT-qPCR supported these results, as it also showed the highest *hSSTR4* expressions in the cerebral cortex and the OB. The mouse *Sstr4* expression was somewhat lower but similar to the *hSSTR4* expression in Chr3 mice, with the exception of the lungs, where it was much higher, and the cerebellum and brain stem, where it was much lower. These findings are in agreement with previous data obtained in large-scale expression studies of both the human and mouse receptors [46,54,55,56]. In both luciferase IVIS and RT-qPCR, U1 and U2 showed some similarities in *hSSTR4* expression for both the pattern and level, suggesting that these two mouse lines carry a copy in the same integration site, but this needs further investigation. Each mouse showed individually different luciferase activity for both the intensity and pattern in the areas of the abdomen and the pelvis. The databases demonstrate variable SST_4_ expression levels (from not detected to moderate) in both human and mouse gastrointestinal and reproductive systems [57,58,59,60,61]. The Chr3 RT-qPCR results showing the relatively high *hSSTR4* expression in the epididymis support this data, but the lower expression in the stomach and the intestine does not. Chr3 had the strongest luciferase signal in the area of the brain (roughly three times as much as observed in U1 and U2), and unlike U1 and U2, we could distinguish and compare the hetero- and homozygous mice. This showed a strong positive association: the double the gene, the double the signal intensity. Therefore, we selected Chr3 as the most useful mouse line and characterized the *hSSTR4* expression by RNAscope.

The tdTomato fluorescence was not detectable in any of the mouse lines either in vivo or in the histological sections, probably due to the low expression level of the *hSSTR4* transgene. TdTomato is usually driven by a strong viral promoter like the human cytomegalovirus or the promoter of a mammalian housekeeping gene like elongation factor-1 alpha, to be expressed at high level for a strong fluorescent signal [62]. Furthermore, while the tdTomato has been considered more tolerant of N-terminal fusion than the mRFP1 it was derived from [63], we observed its fluorescence to be greatly diminished in the luciferase-tdTomato fusion protein compared to the native tdTomato protein (data not shown), probably due to the disrupted folding or tetramerization of the tdTomato [64,65].

The *hSSTR4* had the most prominent signal in the hippocampus (CA1 and CA2) and the cerebral cortex (Pir, S1 and PrL), which corresponds to the mouse and human databases [57,58,59,60,61]. RNAscope in the brain of the Chr3 mice shows *hSSTR4* predominantly to be expressed in *Vglut1*-positive glutamatergic excitatory neurons similarly to the *Sstr4* receptor in wild-type mice, although at a visibly lower expression level. *hSSTR4* was also expressed in GABAergic interneurons in the same regions, whereas, previously, the mouse *Sstr4* mRNA was detected in GABAergic neurons only in the core of the central amygdala. In S1, *hSSTR4* had a higher expression in layer V than in layers II-IV, as opposed to the mouse *Sstr4* expression [46]. In a previous study, in the OB of the wild-type mice, *Sstr4* was expressed in the glomerular layer but not in the granular cell layer [66], whereas, in transgenic mice, the *hSSTR4* was expressed mostly in the granular layer of the OB.

Differences in the expression pattern between human *SSTR4* and mouse *Sstr4* might be due to species differences but can also result from limitations of the humanized mouse model, such as the positional effect of the integration site [51]. Therefore, these differences need to be further investigated.

We concluded that the Chr3 *hSSTR4* mouse line showed measurable *hSSTR4* expression, mainly in excitatory glutamatergic neurons of pain- and mood regulation-related brain regions, with several similarities and, also, some differences compared to the mouse *Sstr4* expression. Therefore, after confirming the receptor function, this transgenic mouse line can be a suitable translational research tool to determine the potential of SST_4_ as an analgesic, antidepressant and anti-inflammatory drug target and to test the SST_4_ agonist candidates during preclinical development.

## 4. Materials and Methods

### 4.1. PiggyBac Transposon Vector

Vector pPBC2 was constructed as follows: The 4 kb sequence upstream of the *hSSTR4* coding region containing the promoter and putative regulatory sequences was obtained in two PCR reactions on human genomic DNA. The first segment was amplified by the forward primer 5′-ATC CTC ATT CAC TAT CCT GGG AAG T-3′ and reverse primer 5′-CCT GGA ATC TTT CCT GTG CCT ACT T-3′ resulting in a 2082-bp-long fragment. The second segment containing the beginning of the *hSSTR4* coding region, and the immediate upstream sequence was amplified by using forward primer 5′TCC TGG AAG CAC TAG CTG TTT ATC A-3′ and reverse primer 5′-TTC ACC AGC GTC TTC TGT CTC ACC-3′, producing a 2580-bp-long fragment. The two PCR fragments were cloned together at the SphI restriction site in the overlapping section, and a XhoI-SmaI fragment of it was incorporated into the construct containing the beginning of the *SSTR4* coding region and 3968 bp upstream sequence. The rest of the *hSSTR4* coding sequence was cloned from vector pcDNA3.1(+)/*SSTR4* (Cat# SSTR400000; Guthrie Research Institute, Sayre, PA, USA) and fused to the previously described fragment at the unique SmaI site in the *hSSTR4* coding sequence. In the final construct, the coding region contains four single-nucleotide polymorphisms (SNP) compared to the human *SSTR4* reference sequence [67]. These high-frequency SNPs are rs3746726 850T > G, rs2567609 897T > C, rs3746728 924C > T and rs2567608 962T > C. The 2645-bp-long downstream genomic region behind the *hSSTR4* coding sequence was obtained by genomic PCR using forward primer 5′-GGA GCC CTT CCC CTA CCC A-3′ and reverse primer 5′-TGG GTA GGG GAA GGG CTC C-3′. The fragment coding the luciferase-tdTomato marker protein was cloned from vector pcDNA(+)/Luc2 = tdT (Cat#32904; Addgene, Watertown, MA, USA). The sequence coding the P2A peptide was constructed from oligonucleotides and inserted between the *hSSTR4* and luciferase-tdTomato sequences. Lox2273 sites and the artificial polyA site was also assembled from oligonucleotides and inserted into the appropriate positions. Finally, the whole construct was inserted into the pB007 PiggyBac vector (Ref# SPB-007; Transposagen, Lexington, KY, USA) to obtain the pPBC2 vector that was used for transgenesis.

### 4.2. Animals

B6.129P2-*Sstr4*^tm1Szo^ (*Sstr4* KO) mice [2,22,23] were used for the transgenesis (both the zygotes and the pseudo-pregnant females), for the early breeding of transgenic mice and, later, as control animals in experiments. Distinct mouse lines were created by breeding, each containing a single copy of the transgene. The proposed name of the humanized *SSTR4* expressing mouse strain is B6.129P2-*Sstr4*^tm1Szo^-TgTn(pb-*SSTR4*-P2A-luc-tdTomato)1Sazo.

Animals were bred and kept in the animal house of the Department of Pharmacology and Pharmacotherapy, University of Pécs Medical School at a temperature of 22 ± 2 °C and a 12-h light–dark cycle. Standard rodent chow and water were provided ad libitum.

### 4.3. Transgenesis

The transgenesis was made in commission by BioTalentum Ltd. (Gödöllő, Hungary). The *hSSTR4* transgene vector (pPBC2) and the super piggyBac transposase mRNA (Ref# SPB-003; Transposagen, Lexington, KY, USA) were microinjected into the pronuclei of 56 zygotes from *Sstr4* KO mice, and the transfected zygotes were implanted into pseudo-pregnant *Sstr4* KO mice that resulted in 16 transfected mice (F0 generation). These mice were genotyped by PCR for both the *hSSTR4* transgene and the vector backbone. Primers for the *hSSTR4* 3′ region: primer TR1: CTT TGC TCA TCC CTC CAT CT and primer TR2: GTC GCT GTG CAT TTA GGA CA (product size: 883 bp). For the *hSSTR4* 5′ region: TR ST4 3: TTG ACG CAT GTG TTT TAT CG and TR ST4 4: ATC CTG GTA CCC ACC CAG AC (product size: 717 bp). For the plasmid origin (vector backbone 1): Cole1Ori-F: ATC GAC GCT CAA GTC AGA GG and Cole1Ori-R: CCG GAT CAA GAG CTA CCA AC (product size: 475 bp). For the Ampicillin resistance gene (vector backbone 2): Backbone ctrl Forward: GTG TCG CCC TTA TTC CCT TT and Backbone ctrl Reverse: AAC TTT ATC CGC CTC CAT CC (product size: 623 bp).

The *hSSTR4* transgene without the plasmid backbone integrated into only 3 female mice from the F0 generation, and all of them were backcrossed with *Sstr4* KO males.

### 4.4. Ligation-Mediated PCR

We followed the protocol and Y-linker sequences from Bryda et al. [52]. Genomic DNA was extracted from tail biopsies using the Thermo Scientific Phire Tissue Direct PCR Master Mix kit (Thermo Fisher Scientific, Waltham, MA, USA). Digestion was performed with different 3′ overhang creator restriction endonucleases separately: TaiI, PstI and HhaI. Three transgene specific nested primers were designed to both 5′ and 3′ end regions of the transgene using Primer-BLAST (NCBI, Rockville Pike, Bethesda MD, USA). The nested primers were oriented to the outward of the transgene, where the Y-linker is expected to be attached, and the primers were arranged accordingly (primer 1 is furthest from the end of the transgene, and primer 3 is the closest). 5′ end specific primers: LM-V1B: CTA TTC AAA TTA ATA AAT AAA CCT CG, LM-V2B: TAA ACC TCG ATA TAC AGA CCG and LM-V3B: CGA TAA AAC ACA TGC GTC AA. 3′ end specific primers: LM-V1: AGC TCC AGC TTT TGT TCC CTT, LM-V2: ACG ACT CAC TAT AGG GCG AAT and LM-V3: GGC GAA TTG GGT ACC GGG. The Y-linker consists of 2 partially complementary oligo DNA fragments: Y-linker A: GTG CAG CCT TGG GTC GCC GTG T/3InvdT/(nonexpendable base end) and Y-linker E: GCA AAC GAT AAA TGC GAG GAC GGT ACA GGC CGA CCC AAG GCT GCA CT [52]. The Y-linker-specific nested PCR primers: Y-linker primer D: GCA AAC GAT AAA TGC GAG GAC GGT and Y-linker primer G: ATG CGA GGA CGG TAC AGG CCG ACC. All primers and the Y-linker were synthesized by Integrated DNA Technologies (IDT; Coralville, IA, USA). The Y-linker was prepared by the annealing of Y-linker A and Y-linker E. The first (single round) PCR was performed with digested DNA, Taq DNA polymerase enzyme (5′ overhang synthesizing tendency) and transgene specific primer 1 (LM-V1 and LM-V1B in separate reactions). The Y-linker was ligated with the product of the first PCR by T4 DNA ligase and then was amplified by 2 consecutive PCRs with nested primer pairs. The second PCR was made with the product of ligation, Y-linker primer D and transgene specific primer 2 (LM-V2 or LM-V2B, matching to the primer 1 used in the first PCR) and the third PCR was made with the product of the second PCR, Y-linker primer G and transgene specific primer 3 (LM-V3 or LM-V3B, matching to the primer 1 and 2 used in the previous PCRs). Each was made with Phire Tissue Direct PCR Master Mix. Products of the third PCR were separated by gel electrophoresis, and all detected bands were isolated using the GeneJET Gel Extraction Kit (Thermo Fisher Scientific, Waltham, MA, USA). The nucleotide sequence of each product of the third PCR was acquired by Sanger sequencing in the commissioned BIOMI Ltd. (Gödöllő, Hungary). The acquired sequences were analyzed by National Center for Biotechnology Information (NCBI) Nucleotide-BLAST. For verification of the identified loci, mouse chromosome specific (near the insertion site of the transgene) primers were designed to be paired with a LM-V3 transgene specific primer: ChrXpr1: AAC TCC TTT ACC CGC TTG CTC for chromosome X, Chr3pr2: CTG GTT CCG AGT CTC TGA GG for chromosome 3 and Chr10pr2: ATA ATG CCC CTG GCA TAG CTT TC for chromosome 10. Chr3 *hSSTR4* mice are since genotyped routinely by LM-V3 forward primer, Chr3pr2 reverse primer and a secondary forward primer Chr3pr3: TCA GGA GCA AGA GAG GAA GA, resulting in PCR products in the size of 527 bp for wild-type chromosomes and 683 bp for the inserted *hSSTR4* transgene, allowing the identification of Chr3 homozygotes and heterozygotes (Figure 3).

### 4.5. Detecting hSSTR4 Expression by In Vivo Optical Imaging of the Luciferase Enzyme and tdTomato

For the in vivo imaging of luciferase, 8–10-week-old male animals were injected with 300 mg/kg D-luciferin sodium salt (Goldbio, St. Louis, MO, USA) intraperitoneally (i.p.) and then anaesthetized with ketamine-xylazine (100 and 5 mg/kg i.p.). The fur was removed with a fine electrical shaver. Bioluminescent imaging was performed 30 min after D-luciferin administration with the IVIS Lumina III imaging system (PerkinElmer, Waltham, MA, USA) with the following settings: exposure time 5 min and, binning 4. The bioluminescent signal of the brain was quantified as a total flux (photons/s) in equal-size regions of interest (ROI) corresponding to the top of the skull. Fluorescent optical imaging of tdTomato expression was also performed with the IVIS Lumina III imaging system. For tdTomato detection, excitation filters of 500, 520, 540 and 560 nm for spectral unmixing and an emission filter of 620 nm, auto exposure and a binning of 2 were used. After imaging, animals were placed onto a heating pad and monitored until they recovered from anesthesia.

### 4.6. Investigating hSSTR4-Linked tdTomato Expression in the Mouse Brain by Confocal Microscopy

After luminescent optical imaging, the animals were deeply anesthetized with an overdose of urethane (2.4 g/kg i.p.) and perfused transcardially with 30 mL of 4% paraformaldehyde in Millonig’s phosphate buffer. Dissected brains were postfixed for 24 h at room temperature (RT), rinsed in 1x phosphate-buffered saline (PBS) and sectioned (by 30 µm) using a vibrating microtome (VT1000S, Leica Biosystems, Wetzlar, Germany). Sections were mounted on Superfrost Ultra Plus slides (Thermo Fisher Scientific, Waltham, MA, USA), air-dried for 3 h at RT and the sections were counterstained with 4′,6-diamidino-2-phenylindole (DAPI) and mounted with ProLong Diamond Antifade Mountant (Thermo Fisher Scientific, Waltham, MA, USA) for confocal imaging.

Fluorescent images of PrL, BLA, S1, CA1, CA2 and Pir, according to Paxinos and Franklin [48], were acquired using an Olympus Fluoview FV-1000 laser scanning confocal microscope and FluoView FV-1000S-IX81 image acquisition software system (Olympus, Tokyo, Japan). The confocal aperture was set to 80 µm. The analog sequential scanning was performed using a 40x objective lens (NA: 0.75). The optical thickness was set to 1 μm, and the resolution was 1024 × 1024 pixels. The excitation time was set to 4 µs per pixel. Virtual colors were selected to depict fluorescent signals: blue for DAPI and red for tdTomato). Images of the two respective channels were stored both individually and superimposed to evaluate the colocalization of fluorescent signals.

### 4.7. Measuring Organ-Specific hSSTR4 Expression by RT-qPCR

Total RNA was extracted from WT (C57BL/6), *Sstr4* KO, Chr3, U1 and U2 8–10-week-old male mice from the following 14 mouse organs: bladder, brain stem, cerebellum, cerebral cortex, epididymis, heart, ileum, kidney, liver, lungs, OB, spleen, stomach and TG. TRI Reagent (Molecular Research Center, Inc., Cincinnati, OH, USA) and Direct-Zol RNA isolation kit (Zymo Research, Irvine, CA, USA) were used for total RNA extraction. The concentration of the RNA samples was measured by a NanoDrop ND-2000 spectrophotometer (NanoDrop Technologies. Wilmington, DE, USA). Genomic DNA contamination in the RNA samples was fully digested by DNase I (Zymo Research, Irvine, CA, USA). Reverse transcription of the RNA samples was made by Maxima First Strand cDNA Synthesis Kit for RT-qPCR (Thermo Scientific, Waltham, MA, USA). Incubation for DNA digestion, DNase I heat inactivation and reverse transcription PCR were performed in a Biometra TAdvanced Twin 48/48 G^3^ (Analitik Jena, Jena, Germany). Quantitative PCR was performed in an Applied Biosystems QuantStudio 5 Real-Time PCR System (Thermo Fisher Scientific, Waltham, MA, USA). Each reaction contained 20 ng of cDNA sample, 1X Luminaris Color HiGreen Low ROX qPCR Master Mix (Thermo Scientific, Waltham, MA, USA), 1 µM of each primers and additional nuclease-free water to a total reaction volume of 20 µL. The following primer pairs were used: for the *hSSTR4* cDNA: hSSTR4 new F: TGG AAG GTG CTG GAG GT and hSSTR4 new R: GTT CTG GTT GCA GGG CTT; for the mouse *Sstr4* cDNA: mSSTR4gfor: GCC CTG GTC ATC TTC GTG AT and mSSTR4grev: ATG AAG AGC TCA TCG GCG AC and for the reference gene beta actin (*Actb*) cDNA: mBACTIN F: GTC GAG TCG CGT CCA CC and mBACTIN R: GTC ATC CAT GGC GAA CTG for endogenous control. These primers were tested on KO mice (data not shown) and RT-qPCR amplification and the melting curve analysis indicated that both the human *SSTR4* and mouse *Sstr4* primers are specific to their respective genes. Water control was used to check the reactions for contamination. Real-time qPCR was performed under the following conditions: 95 °C for 10 min, followed by 40 cycles of 95 °C for 30 s, 60 °C for 30 s and 72 °C for 1 min. Each reaction was carried out in a duplicate. Signal specificity was ensured by melt curve analysis. Relative expression ratios were calculated using the ΔCt method and linearized by 2^-ΔCt, in which the *hSSTR4* expression level was compared to the *Actb* reference gene.

### 4.8. Characterizing hSSTR4 Expressing Neurons in the Mouse Brain by RNAscope In Situ Hybridization

RNAscope ISH were performed on 3 to 4-month-old male *Sstr4* KO and *hSSTR4* Chr3 Hom mice (*n* = 4). Animals were deeply anesthetized with an overdose of urethan (2.4 g/kg) and perfused transcardially with 30 mL of 4% paraformaldehyde in Millonig’s phosphate buffer. Dissected brains were postfixed for 72 h at 4 °C, rinsed in 1x PBS and sectioned (by 30 µm) using a vibrating microtome (VT1000S, Leica Biosystems, Wetzlar, Germany). Sections were stored in 1x PBS with 0.01% Na-azide (Merck KGaA, Darmstadt, Germany).

RNAscope assay was performed on 30-µm-thick vibratome-sliced brain sections using RNAscope Multiplex Fluorescent Reagent Kit v2 (Advanced Cell Diagnostics, Newark, CA, USA), according to the manufacturer’s protocols. Tissue pretreatment was performed by treatment with 1 *v/v*% H_2_O_2_ solution in PBS for 30 min. After PBS washes, sections were mounted on Superfrost Ultra Plus slides (Thermo Fisher Scientific, Waltham, MA, USA). Sections were air-dried for 3 h at RT and incubated at 60 °C for 60 min. After 2 × 10-min washes in Milli-Q (MQ) water, slides were incubated in 10% neutral buffered formalin solution (NBF, Merck KGaA) at 4 °C for 2 min. After 3 × 10 min MQ water rinses, sections were digested in proteinase K solution at 37 °C for 15 min (0.01 mg/ml proteinase K (EO0491, Thermo Fisher Scientific, Waltham, MA, USA) in 0.1 M Tris/HCl, pH = 8 and 0.05 M EDTA, pH = 8 buffer. After rinsing in MQ water, slides were treated with 10% NBF at 4 °C for 2 min, followed by 3 × 10-min washes in MQ water. Sections were hybridized with probes specific to human *SSTR4* and mouse *Sstr4*, *Vglut1* and *Gad1*, in parallel with RNAscope 3-plex mouse positive and negative control probes. Signal amplification, channel development and mounting were performed according to the manufacturer’s protocols. Sections were counterstained with 4′,6-diamidino-2-phenylindole (DAPI) and mounted with ProLong Diamond Antifade Mountant (Thermo Fisher Scientific, Waltham, MA, USA) for confocal imaging. Fluorescent images of PrL, BLA, S1, CA1, CA2 and Pir, according to Paxinos and Franklin [68], were acquired using an Olympus Fluoview FV-1000 laser scanning confocal microscope and FluoView FV-1000S-IX81 image acquisition software system (Olympus, Tokyo, Japan). The confocal aperture was set to 80 µm. The analog sequential scanning was performed using a 40x objective lens (NA: 0.75). The optical thickness was set to 1 μm, and the resolution was 1024 × 1024 pixels. The excitation time was set to 4 µs per pixel. Virtual colors were selected to depict the fluorescent signals: blue for DAPI, green for fluorescein (*Vglut1* mRNA), red for Cyanine 3 (*hSSTR4* and *mSstr4* mRNA) and white for Cyanine 5 (*Gad1* mRNA). Images of the respective four channels were stored both individually and superimposed to evaluate the co-localization of fluorescent signals.

## Data Availability

The data presented in this study are available on request from the corresponding author.

## Abbreviations

BLA	basolateral nucleus of the amygdala
BMA	basomedial nucleus of the amygdala
CA1	Cornu Ammonis 1 region of the hippocampus
CA2	Cornu Ammonis 2 region of the hippocampus
Ct value	threshold cycle value (in RT-qPCR)
*dabP*	gene of the bacterial D site of albumin promoter (albumin D-box) binding protein
DAPI	4′,6-diamidino-2-phenylindole, a blue-fluorescent DNA stain
DNA	deoxyribonucleic acid
*FOXA2*	gene of the human forkhead box A2
GABA	gamma-aminobutyric acid
*Gad1*	gene of the mouse glutamate decarboxylase 1
Het	heterozygote/heterozygous, carrying different alleles of the gene
Hom	homozygote/homozygous, carrying identical alleles of the gene
*hSSTR4*	gene of human somatostatin receptor subtype 4
i.p.	intraperitoneally
KO	knock-out, an artificially created nonfunctional variant of the gene
LM-PCR	ligation-mediated polymerase chain reaction
mRNA	messenger ribonucleic acid
NCBI	National Center for Biotechnology Information
OB	olfactory bulb
*Otol1*	gene of the mouse otolin 1
PB	piggyBac, a type of transposon system
PBS	phosphate-buffered saline
pcDNA	plasmid cloning DNA
PCR	polymerase chain reaction
Pir	piriform cortex
*Polr2a*	gene of the mouse RNA polymerase II subunit A
polyA	polyadenylation signal sequence
*Ppib*	gene of the mouse peptidylprolyl isomerase B
PrL	prelimbic cortex
RT	room temperature
RT-qPCR	reverse transcription quantitative polymerase chain reaction
S1	primary somatosensory cortex
*Sis*	gene of the mouse sucrase-isomaltase
SNP	single-nucleotide polymorphism
SST_1-5_	somatostatin receptor subtype 1-5
*Sstr4*	gene of mouse somatostatin receptor subtype 4
SV	seminal vesicle
tdTomato	tandem dimer Tomato, a red fluorescent reporter protein
TG	trigeminal ganglion
*THBD*	gene of the human thrombomodulin
*Ubc*	gene of the mouse ubiquitin C
*Vglut1*	gene of the mouse vesicular glutamate transporter 1
WT	wild type
